# Supplementary material for: Therapeutic gene editing in CD34+ hematopoietic progenitors from Fanconi anemia patients
Source: EMBO Mol Med. 2017 Sep 12;9(11):1574–88. doi: 10.15252/emmm.201707540 (PMC5666315; doi:10.15252/emmm.201707540)
Supplement: Supplementary file 1 — Expanded View Figures PDF [file EMMM-9-1574-s001.pdf]

Expanded View Figures

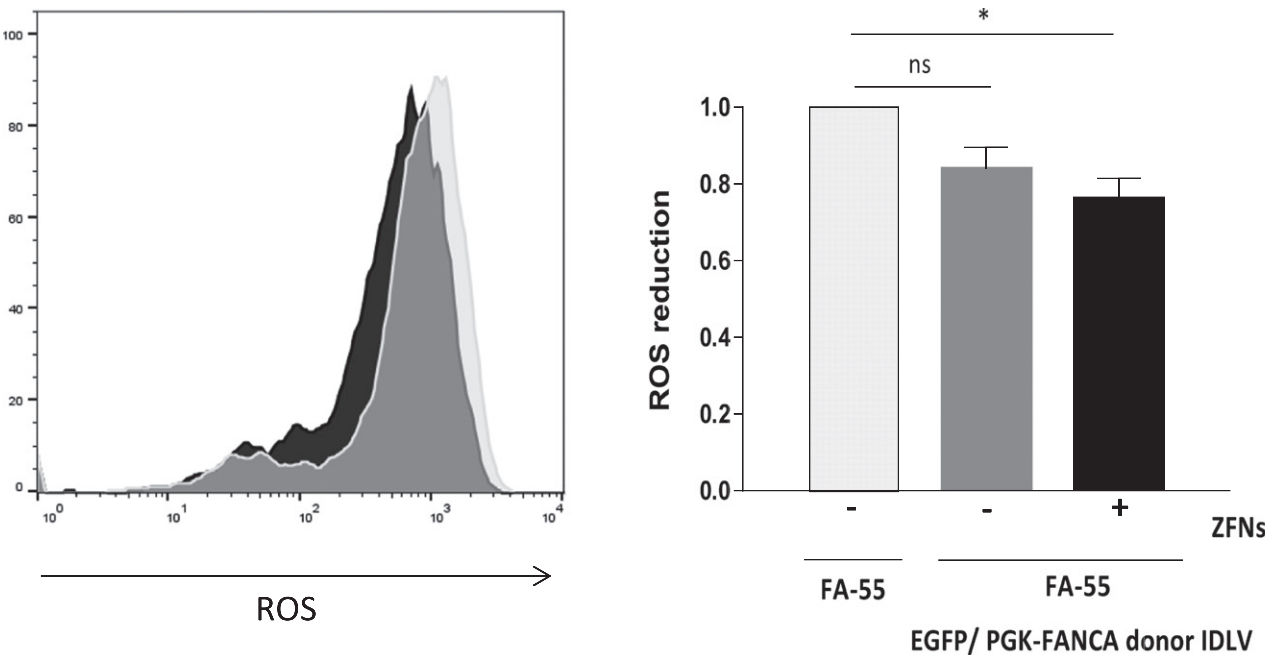

Figure EV1. ROS production in gene-edited LCLs from FA-A patients.

ROS production was measured in the LCL from patient FA-55 targeted with the PGK-*FANCA*/Puro^R donor IDLV in the presence (+) or absence (−) of ZFNs and compared with untreated FA-A LCLs. Left panel: representative ROS production flow cytometry histogram. Right panel: Quantification of ROS production in three different experiments. Mean values and SD are shown ($n = 3$). One-way ANOVA followed by Bonferroni's *post hoc* test was conducted. *$P \leq 0.05$.

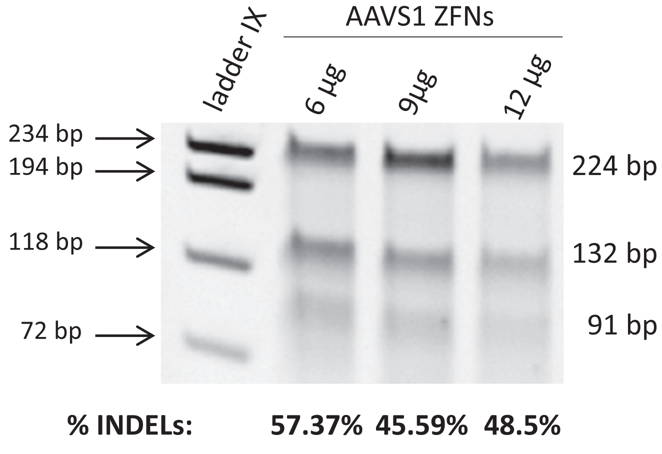

Figure EV2. Evaluation of the efficiency of ZFNs mRNA to target CD34+ cells.

Surveyor assay using increasing doses of *AAVS1*-ZFNs mRNA (6–12 µg). Surveyor bands and the INDELs percentage are also indicated.

 © 2017 The Authors

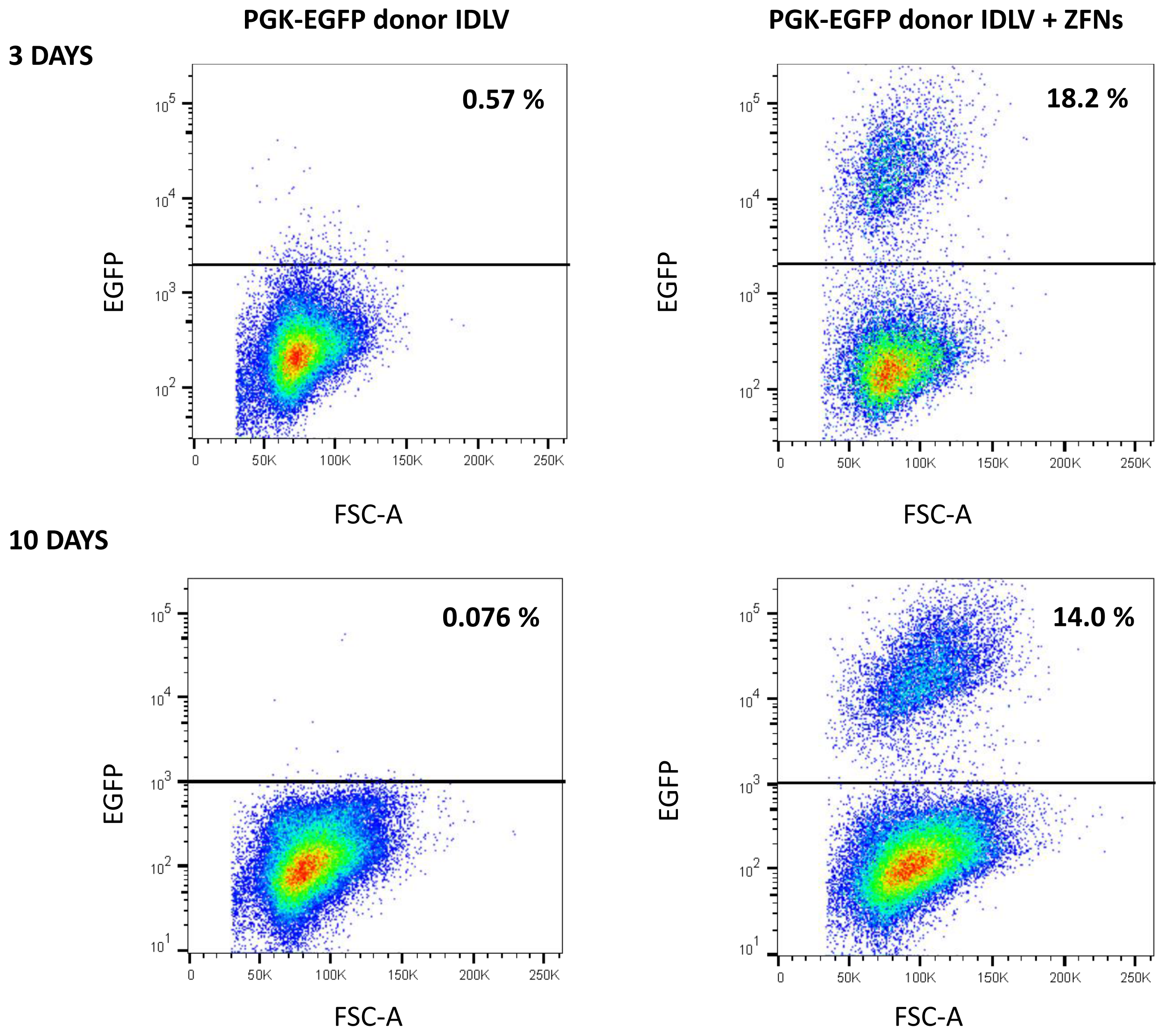

Figure EV3. Flow cytometry analysis of EGFP expression in $CD34^+$ cells after gene editing.

Analysis of $EGFP^+$ cells 3 and 10 days after gene-targeting protocol when the PGK-EGFP IDLV donor and *AAVS1*-ZFNs mRNA or only the PGK-EGFP IDLV donor was used.

© 2017 The Authors

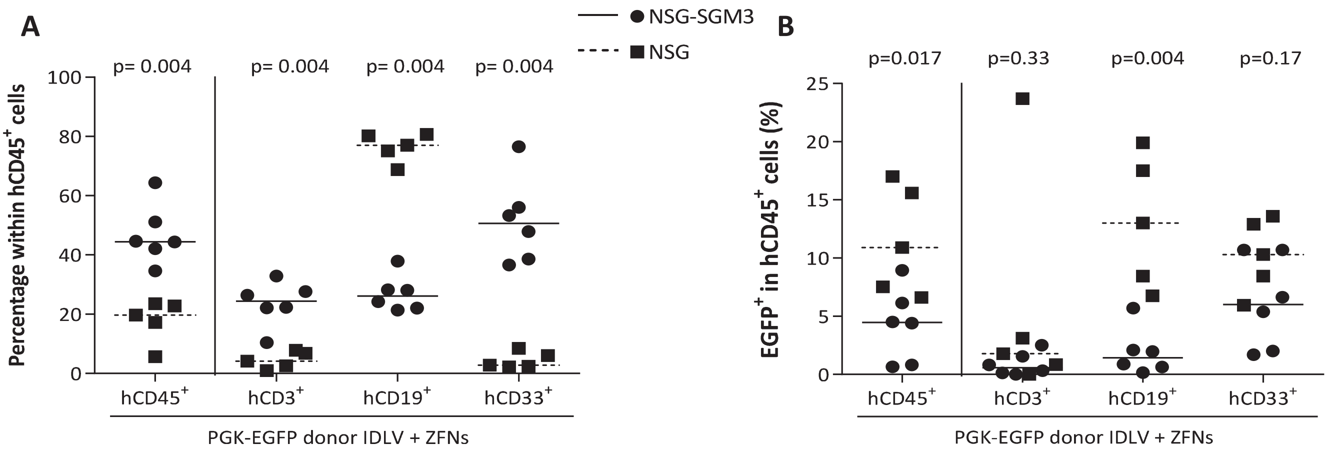

Figure EV4. Engraftment of gene-edited human cells in the spleen of immunodeficient mice 90 days post-transplantation.

A Analysis of human engraftment and the different hematopoietic subpopulations in the spleen of mice transplanted with gene-edited cells. CD3 for T cells, CD19 for B cells and CD33 for myeloid cells.

B Analysis of the percentage of $EGFP^+$ cells in the different hematopoietic subpopulations detected in the spleen 90 days post-transplantation.

Data information: Squares and circles represent data corresponding to individual NSG and NSG-SGM3 mice, respectively. Dashed and solid lines indicate median values in NSG mice and NSG-SGM3 mice, respectively. Statistical analyses were conducted using Mann–Whitney test. n = 5 NSG mice and n = 6 NSG-SGM mice.

© 2017 The Authors

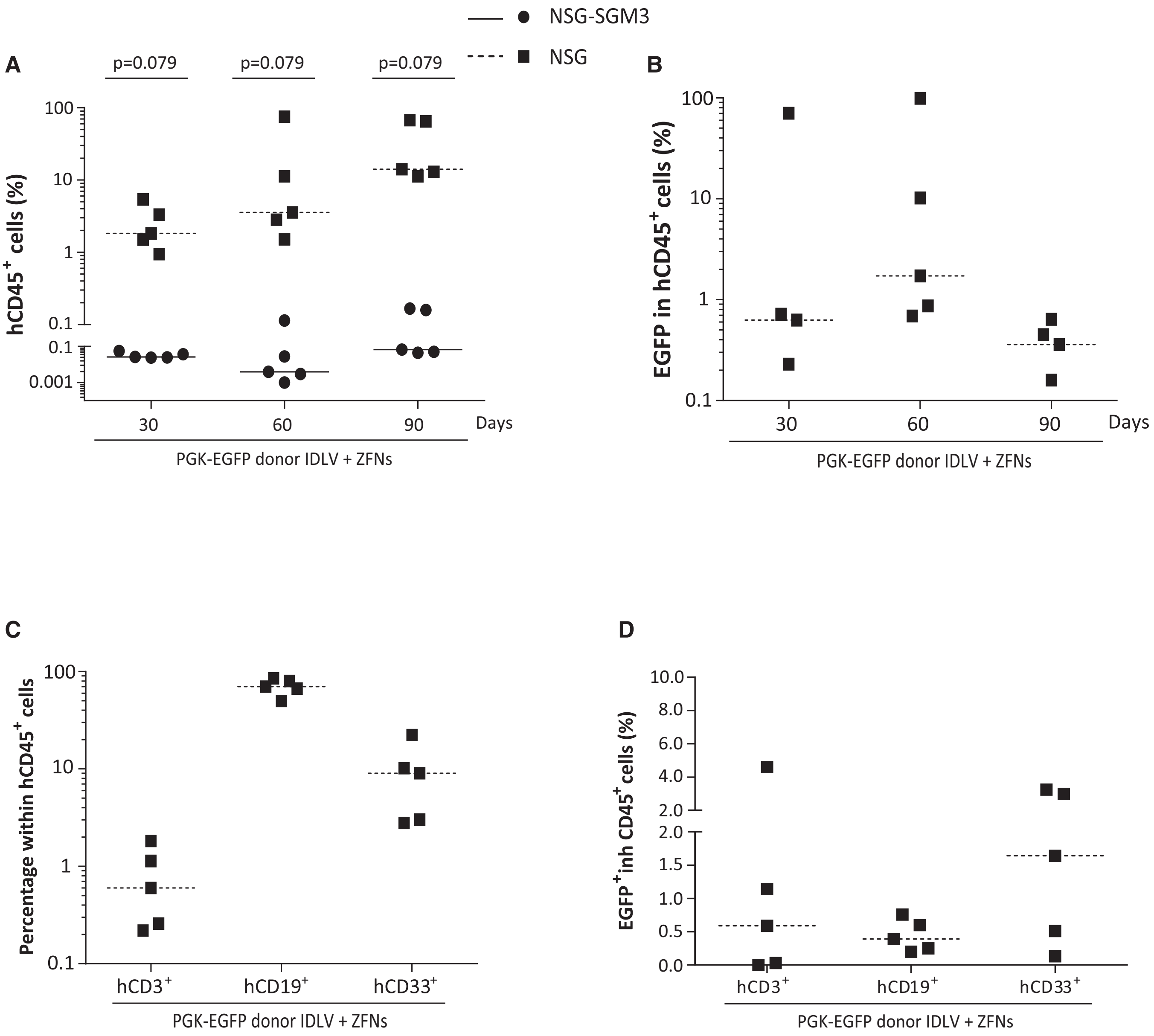

Figure EV5. Long-term engraftment capacity of gene-edited CD34+ cells in secondary mice.

A HSCs from each primary mouse were transplanted in secondary mice and human engraftment was measured as the % of hCD45+ cells.
B Percentage of EGFP+ cells in the human CD45+ population at 30, 60, and 90 days post-transplantation in secondary mice transplanted from primary NSG mice.
C Multilineage BM reconstitution in secondary NSG derived mice was evaluated at the final point by flow cytometry using CD3 antibody for T cells, CD19 for B cells and CD33 for myeloid cells.
D Percentage of EGFP+ cells in the different subpopulations of the BM.

Data information: Squares: Cells from NSG primary mice were transplanted in NSG secondary mice. Circles: Cells from NSG-SGM3 primary mice were transplanted in NSG secondary mice. In all cases, one mouse was transplanted to one mouse. Data are represented as median values (dashed lines indicate median value in NSG mice, and solid lines median value in NSG-SGM3 mice). Statistical analysis was conducted using Mann–Whitney test. n = 5 NSG mice and n = 5 NSG-SGM3 mice.

© 2017 The Authors

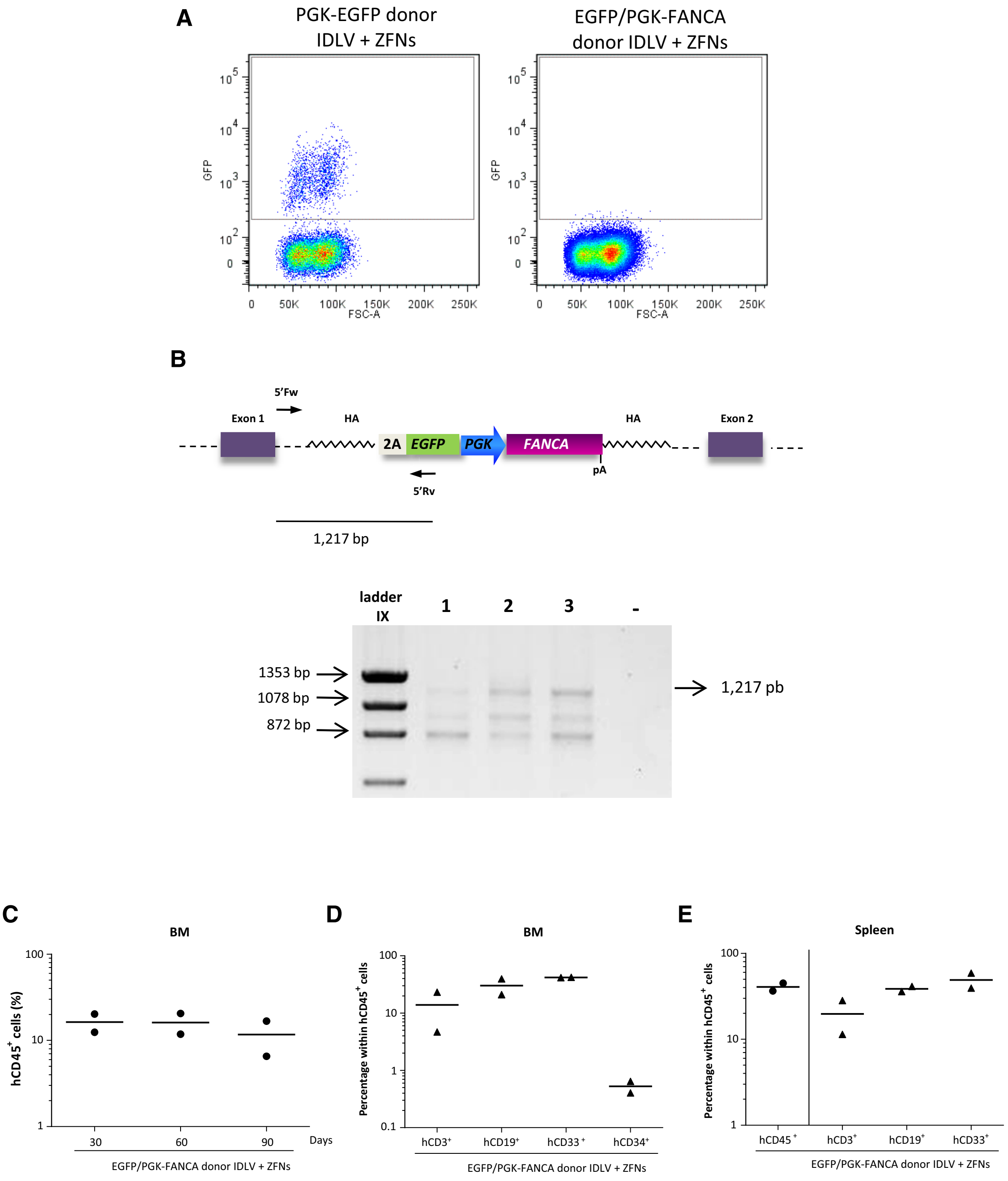

Figure EV6. Gene targeting of the therapeutic EGFP/PGK-*FANCA* donor in the *AAVS1* locus of HD CD34+ cells.

A Representative flow cytometry dot plot of CD34+ cells targeted with the PGK-EGFP donor or EGFP/PGK-*FANCA* donor together with AAVS1-ZFNs at day 10 after targeted integration.

B Upper panel: Schematic representation of gene-targeted *AAVS1* locus with PGK-EGFP donor. The arrows represent the primers used to evaluate the specific integration of the donor in the *AAVS1* site. Bottom panel: Integration analysis by PCR of different pools of individual colonies that presented more than 0.5 copies of EGFP/cell determined by qPCR analysis. –: negative control.

C Analysis of human engraftment (% hCD45+ cells) in BM of NSG-SGM3 recipients transplanted with gene-targeted cells using donor EGFP/PGK-*FANCA*. Analysis was conducted 30, 60, and 90 days post-transplantation. Horizontal lines represent the median. $n = 2$.

D Analysis of the percentage of EGFP+ cells in the different hematopoietic subpopulations detected in the BM 90 days post-transplantation: CD3 for T cells, CD19 for B cells, CD33 for myeloid cells, and CD34 for hematopoietic stem and progenitor cells were used. Horizontal lines represent the median. $n = 2$.

E Analysis of the percentage of EGFP+ cells in the different hematopoietic subpopulations detected in the spleen 90 days post-transplantation. Horizontal lines represent the median. $n = 2$.

 © 2017 The Authors
