# Supplementary material for: Therapeutic gene editing in CD34+ hematopoietic progenitors from Fanconi anemia patients
Source: EMBO Mol Med. 2017 Sep 12;9(11):1574–88. doi: 10.15252/emmm.201707540 (PMC5666315; doi:10.15252/emmm.201707540)
Supplement: Supplementary file 2 — Table EV1 [file EMMM-9-1574-s002.docx]

| **LCL ID** | **Mutation 1** | | **Mutation 2** | | **Reference** |
| --- | --- | --- | --- | --- | --- |
|  | **DNA** | **Protein** | **DNA** | **Protein** |  |
| **FA-55** | c.295C>T | Q99X | c.295C>T | Q99X | (Castella et al, 2011) |
| **FA-56** | c.233_236del TTGA | 178TfsX16 | c.3913C>T | L1305F |  |
| **FA-122** | c.295C>T | Q99X | c.295C>T | Q99X |  |
| **FA-378** | ex 18-20del | No protein | c.1873G>C | C625S |  |

**Table EV1:** Mutations previously described in LCLs from FA-A patients.
