# Supplementary material for: Therapeutic gene editing in CD34+ hematopoietic progenitors from Fanconi anemia patients
Source: EMBO Mol Med. 2017 Sep 12;9(11):1574–88. doi: 10.15252/emmm.201707540 (PMC5666315; doi:10.15252/emmm.201707540)
Supplement: Supplementary file 3 — Table EV2 [file EMMM-9-1574-s003.docx]

**Table EV2:** Primers used to detect the specific integration of the HR cassette in the *AAVS1* locus.

| **Primer** | **Region** |  | **Sequence (5’ to 3’)** | **Tm (°C)** | **PCR product size (bp)** |
| --- | --- | --- | --- | --- | --- |
| PGK-EGFP donor IDLV | 5’ | Fw | AACTCTGCCCTCTAACGCTGC | 59 | 1041 |
|  |  | Rv | ACGTGAAGAATGTGCGAGACCCAG | 59 |  |
|  | 3’ | Fw | AACGGGGATGCAGGGGAACG | 59 | 997 |
|  |  | Rv | TTGCATCGCATTGTCTGAGTAGG | 59 |  |
| PGK-*FANCA*/Puro^R^ donor IDLV | 5’ | Fw | AACTCTGCCCTCTAACGCTGC | 59 | 1041 |
|  |  | Rv | ACGTGAAGAATGTGCGAGACCCAG | 59 |  |
|  | 5’ | Fw | acttcccctcttccgatgtt | 54 | 1230 |
|  |  | Rv | gacgtgaagaatgtgcgaga | 54 |  |
| EGFP/PGK-*FANCA* donor IDLV | 5’ | Fw | AACTCTGCCCTCTAACGCTGC | 59 | 1217 |
|  |  | Rv | tggtgcagatgaacttcaggg | 59 |  |
|  | 3’ | Fw | AACGGGGATGCAGGGGAACG | 59 | 997 |
|  |  | Rv | TTGCATCGCATTGTCTGAGTAGG | 59 |  |
|  | 3’ | Fw | AAAGCTCGTCTTTTTCTGCTGCAGT | 59 | 1332 |
|  |  | Rv | TTGCATCGCATTGTCTGAGTAGG | 59 |  |

PCRs conditions: 10 min at 95°C, 40 cycles of 45 s at 95°C, 45 s at Tm, 2 min at 72°C, and one final step for 10 min at 72°C.
