# Supplementary material for: Therapeutic gene editing in CD34+ hematopoietic progenitors from Fanconi anemia patients
Source: EMBO Mol Med. 2017 Sep 12;9(11):1574–88. doi: 10.15252/emmm.201707540 (PMC5666315; doi:10.15252/emmm.201707540)
Supplement: Supplementary file 4 — Table EV3 [file EMMM-9-1574-s004.docx]

**Table EV3:** Primers used to detect the possible off-targets sites of *AAVS1*-ZFNs

| **Primer** | | **Sequence (5’ to 3’)** | **Tm (°C)** | **PCR product size (bp)** |
| --- | --- | --- | --- | --- |
| CHRAC1 | Fw | ATAGCATTGCCACCAGCAGTGTGT | 62 | 270 |
|  | Rv | CAGCTATTCGGGAGGCTGAG | 62 |  |
| ATRNL1 | Fw | GAAGGTGGTGCTTAACCTTCCAG | 62 | 270 |
|  | Rv | GGCACTGTATGGCAAAGGACC | 62 |  |
| BEGAIN | Fw | ACCTGGACCTTGGCCACGACA | 62 | 270 |
|  | Rv | CTTAGTGCCGTGGGTGAGTG | 62 |  |
| LINC00548 | Fw | CCTGTGGTGAGATAGCACCTCTAA | 62 | 270 |
|  | Rv | TATCTGGGGTGGCCCACAAAAG | 62 |  |
| H19 | Fw | AACCTCAGGCACCCCTCAAC | 62 | 270 |
|  | Rv | GTCCTCCCTTTTAGCATCTACCAG | 62 |  |
