# Supplementary figures and images for: Therapeutic gene editing in CD34+ hematopoietic progenitors from Fanconi anemia patients
Source: EMBO Mol Med. 2017 Sep 12;9(11):1574–88. doi: 10.15252/emmm.201707540 (PMC5666315; doi:10.15252/emmm.201707540)

## Slide 1
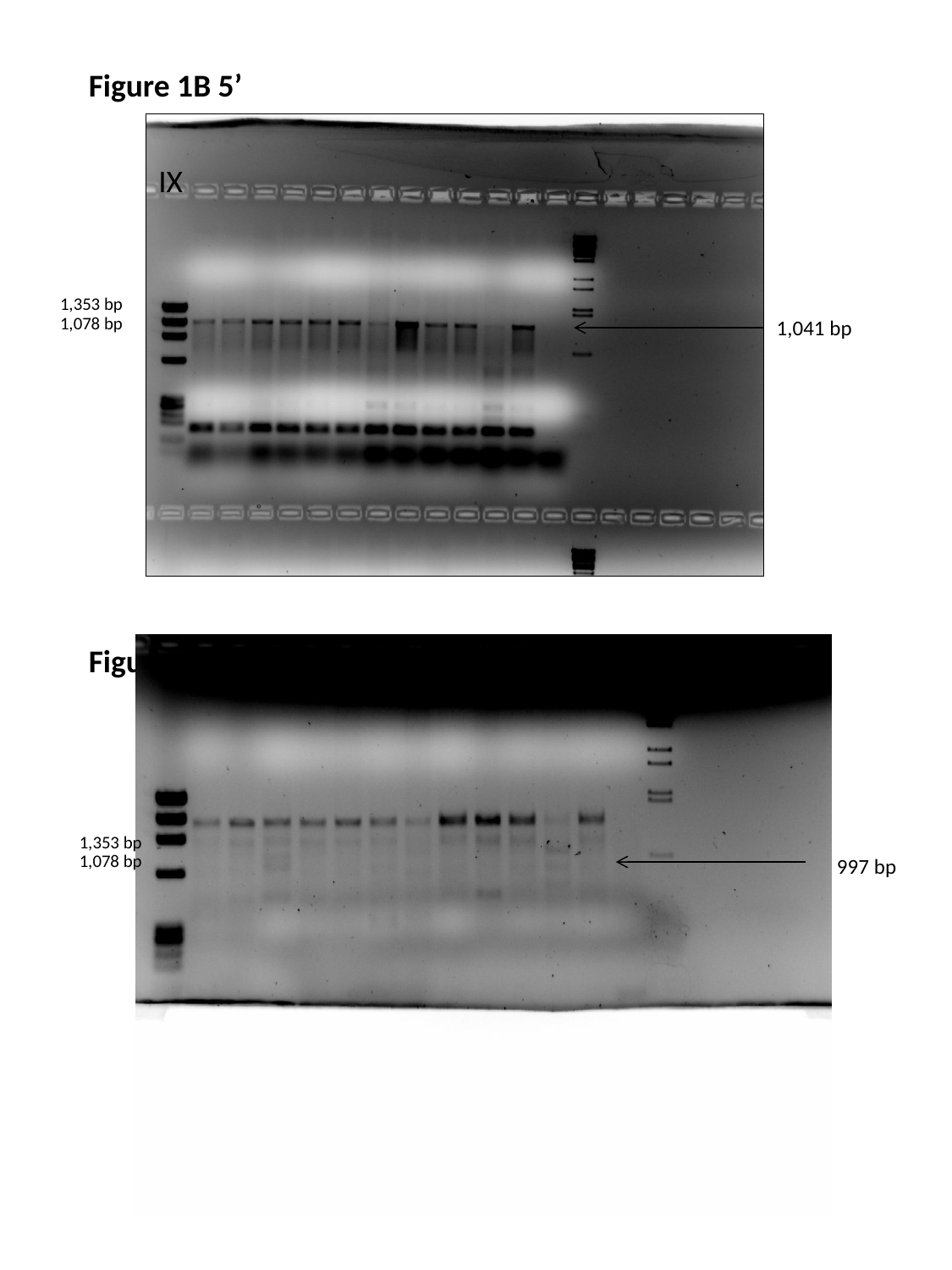

Figure 1B 5’
IX
1,353 bp
1,078 bp
1,041 bp
Figure 1B 3’
1,353 bp
1,078 bp
997 bp

Supplement: Supplementary file 6 — Source Data for Figure 1 [file EMMM-9-1574-s005.pptx]

## Slide 1
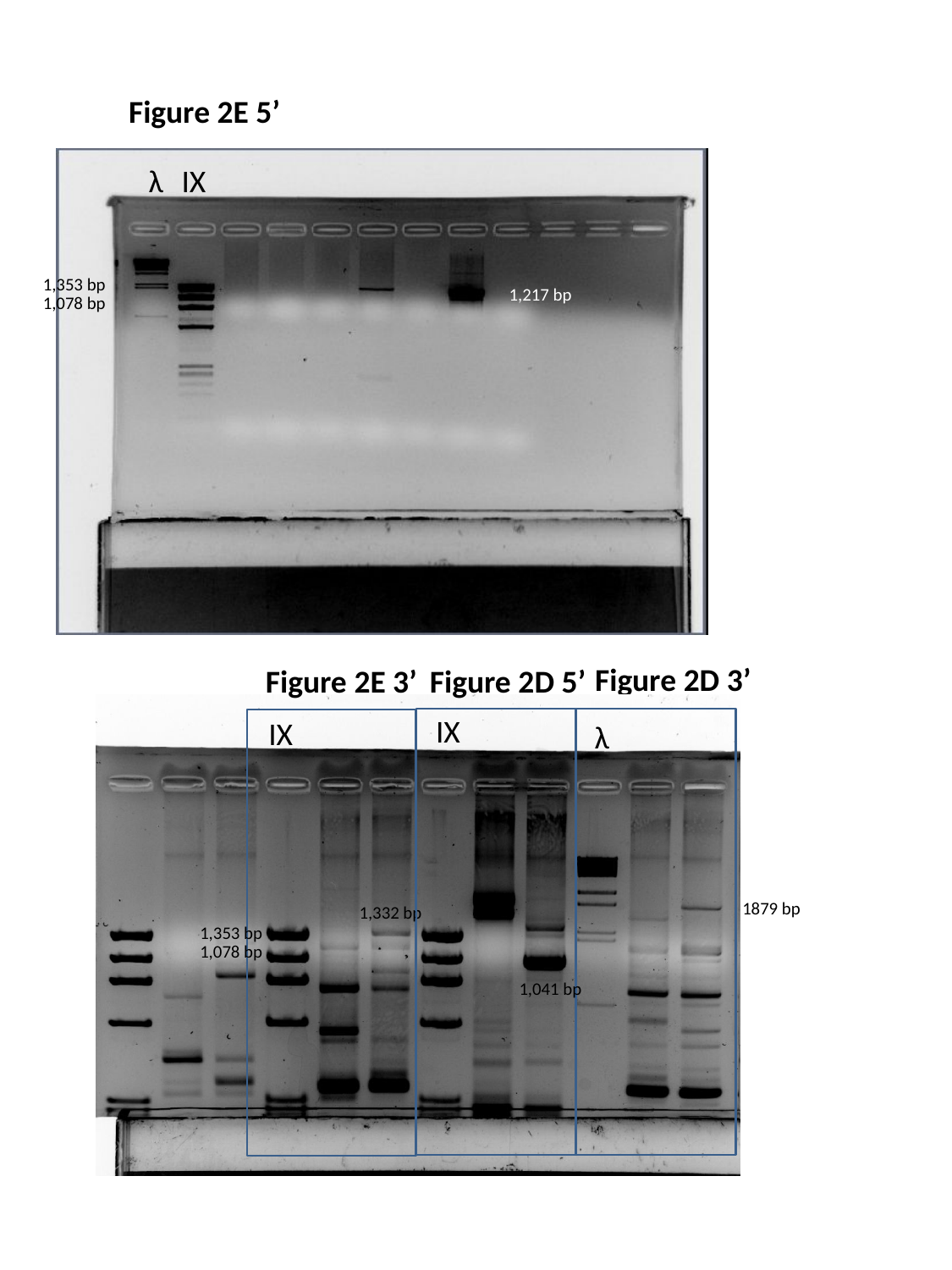

Figure 2E 5’
λ
IX
1,353 bp
1,217 bp
1,078 bp
Figure 2D 3’
Figure 2D 5’
Figure 2E 3’
IX
IX
λ
1879 bp
1,332 bp
1,353 bp
1,078 bp
1,041 bp

Supplement: Supplementary file 7 — Source Data for Figure 2 [file EMMM-9-1574-s006.pptx]

## Slide 1
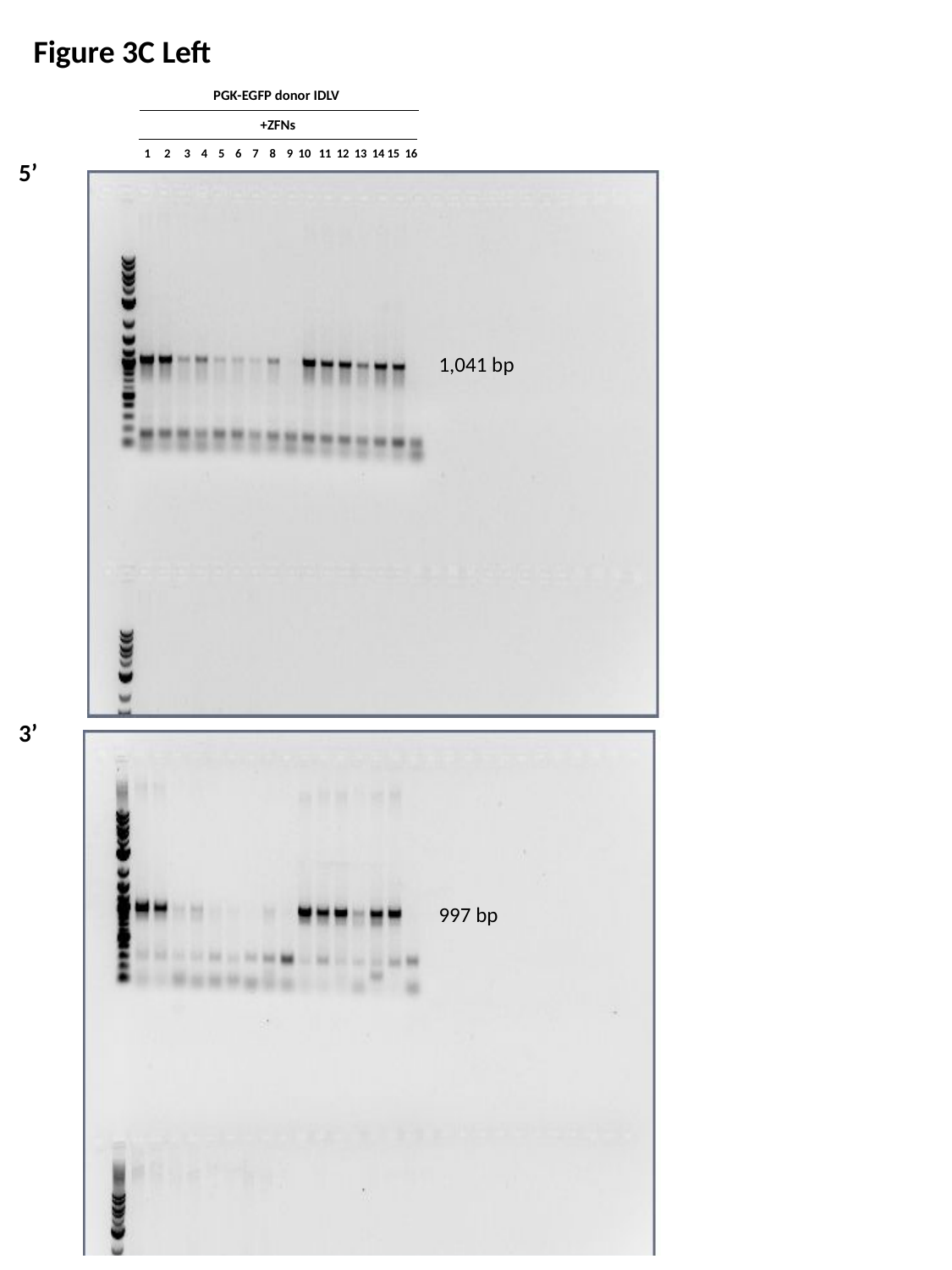

Figure 3C Left
PGK-EGFP donor IDLV
+ZFNs
1 2 3 4 5 6 7 8 9 10 11 12 13 14 15 16
5’
1,041 bp
3’
997 bp

## Slide 2
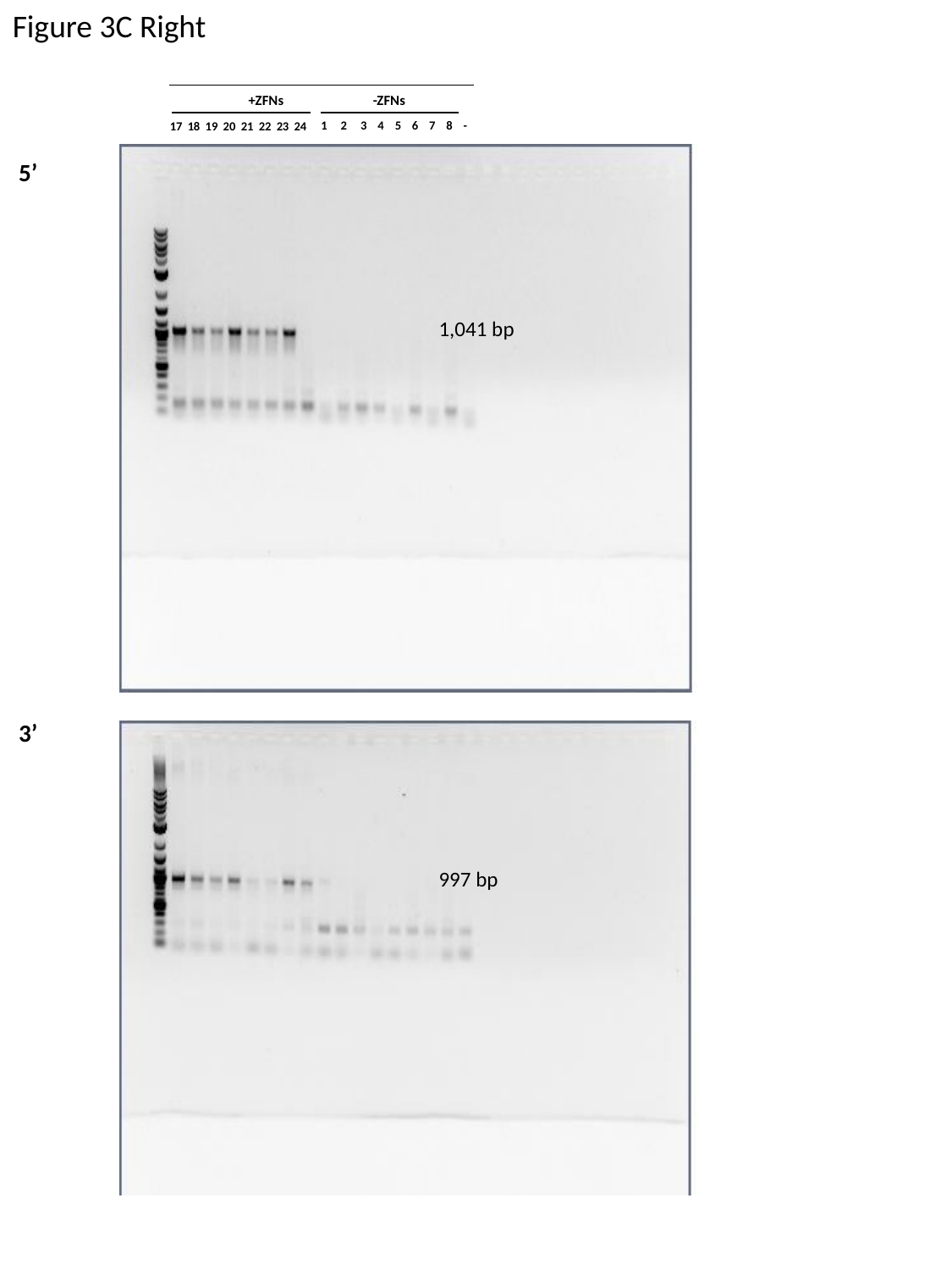

Figure 3C Right
+ZFNs
-ZFNs
1 2 3 4 5 6 7 8 -
17 18 19 20 21 22 23 24
5’
1,041 bp
3’
997 bp

Supplement: Supplementary file 8 — Source Data for Figure 3 [file EMMM-9-1574-s007.pptx]
