# Supplementary material for: Therapeutic gene editing in CD34+ hematopoietic progenitors from Fanconi anemia patients
Source: EMBO Mol Med. 2017 Sep 12;9(11):1574–88. doi: 10.15252/emmm.201707540 (PMC5666315; doi:10.15252/emmm.201707540)
Supplement: Supplementary file 9 — Source Data for Figure 4 [file EMMM-9-1574-s008.pptx]

## Slide 1
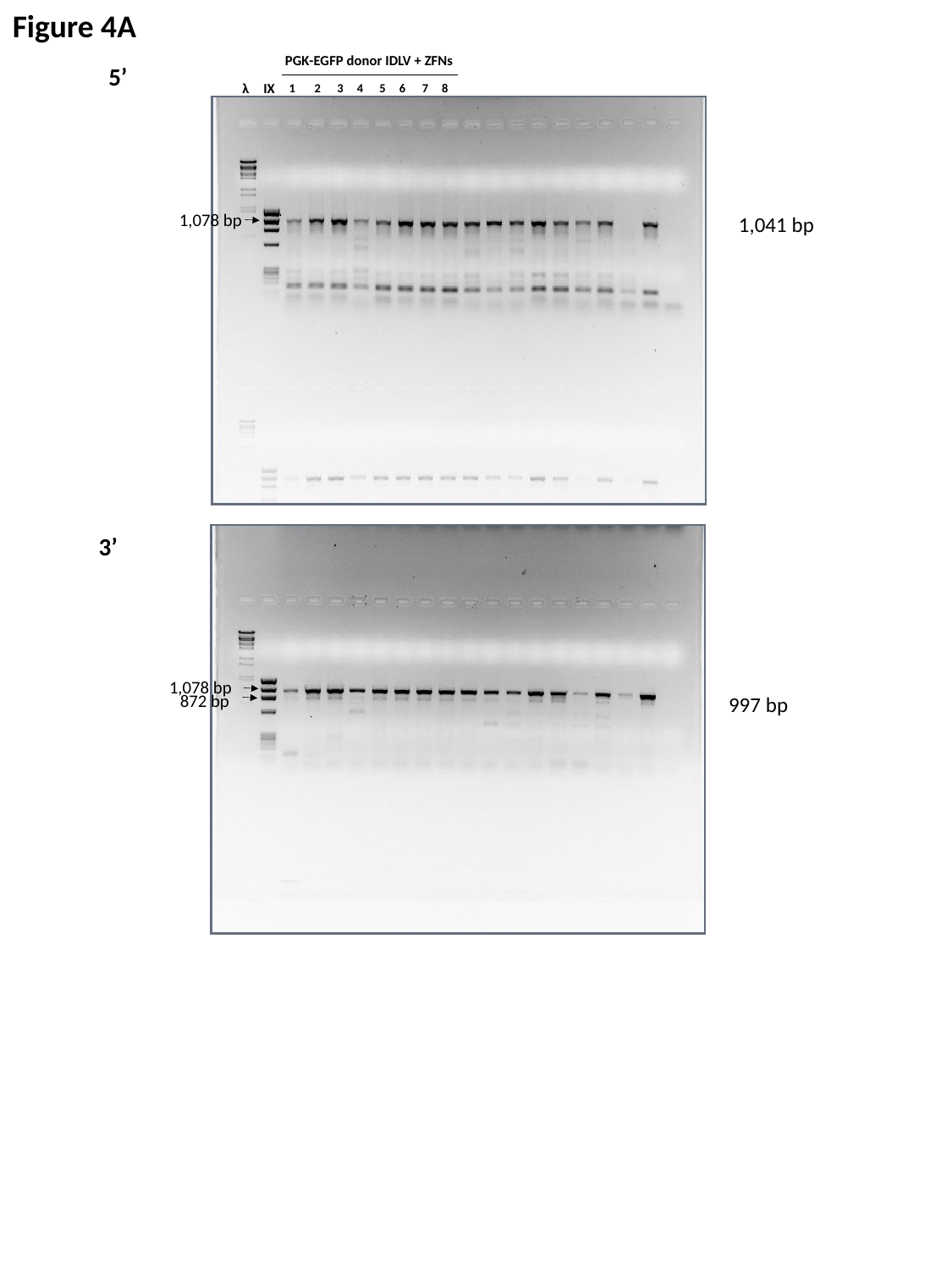

Figure 4A
PGK-EGFP donor IDLV + ZFNs
5’
λ
IX
1 2 3 4 5 6 7 8
1,078 bp
1,041 bp
997 bp
3’
1,078 bp
872 bp
997 bp

## Slide 2
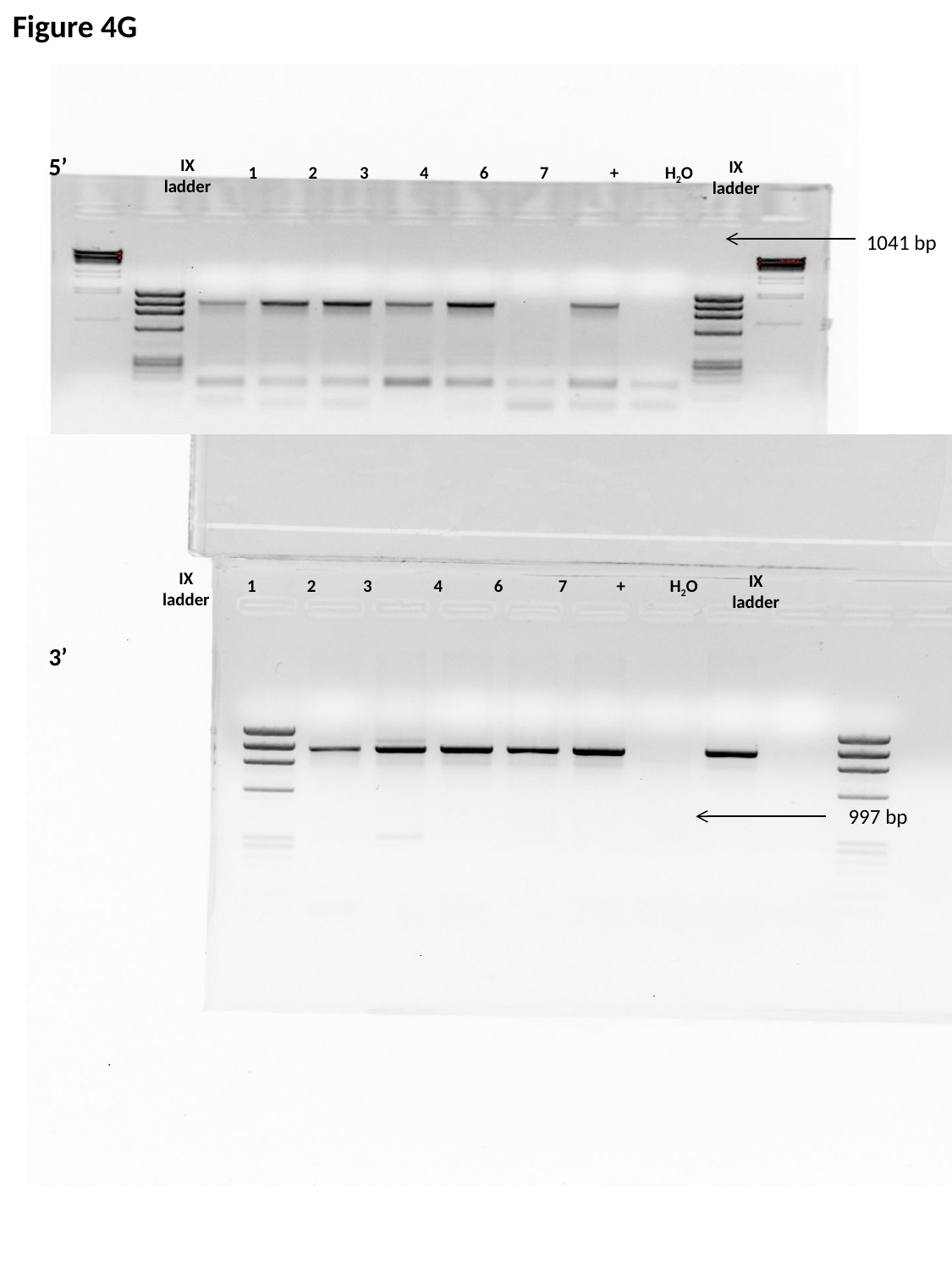

Figure 4G
5’
IX ladder
IX ladder
1
2
3
4
6
7
 +
H2O
1041 bp
IX ladder
IX ladder
1
2
3
4
6
7
 +
H2O
3’
997 bp

## Slide 3
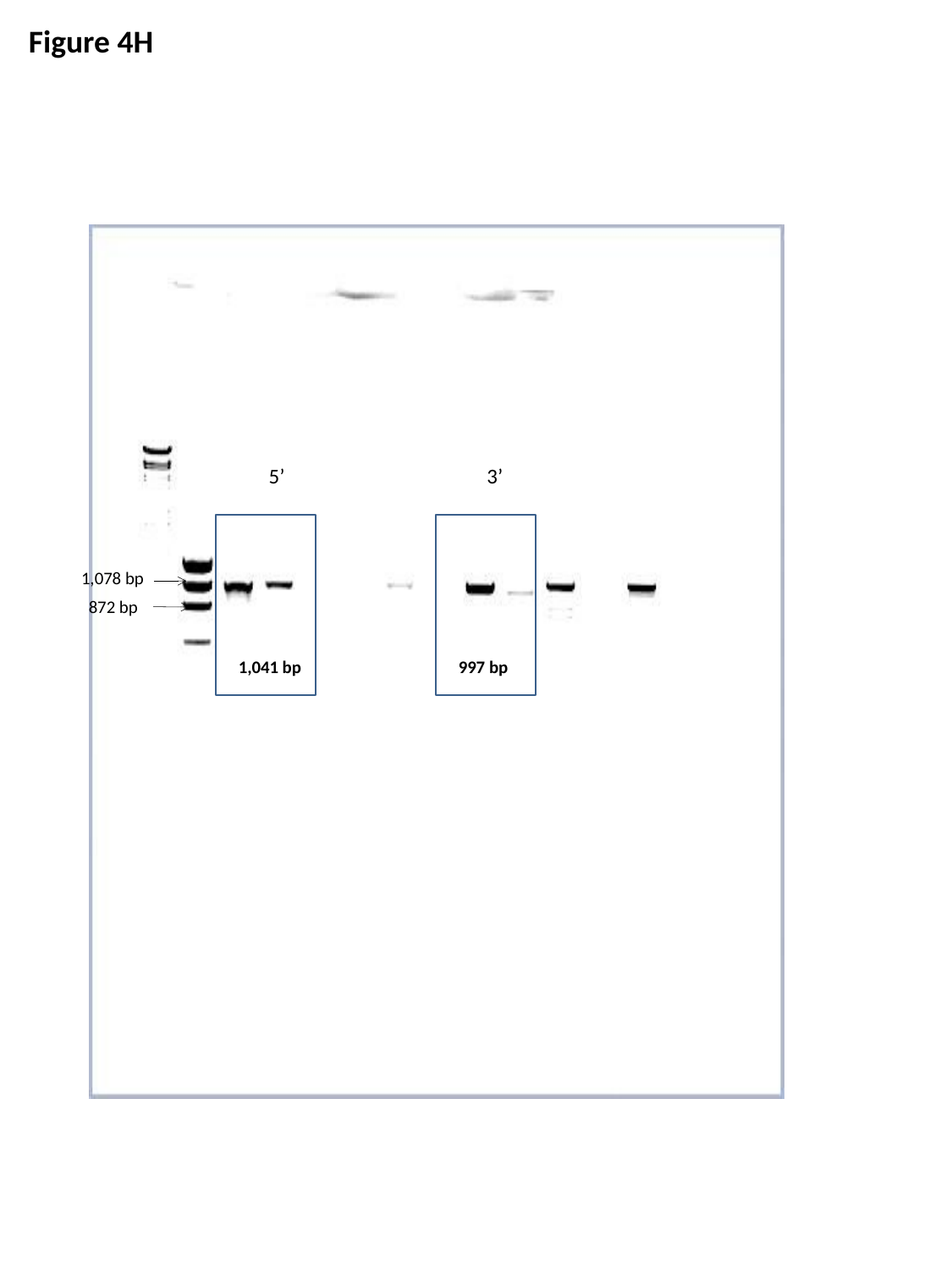

Figure 4H
5’
3’
1,078 bp
872 bp
1,041 bp
997 bp
