# Supplementary material for: Therapeutic gene editing in CD34+ hematopoietic progenitors from Fanconi anemia patients
Source: EMBO Mol Med. 2017 Sep 12;9(11):1574–88. doi: 10.15252/emmm.201707540 (PMC5666315; doi:10.15252/emmm.201707540)
Supplement: Supplementary file 10 — Source Data for Figure 5 [file EMMM-9-1574-s009.pptx]

## Slide 1
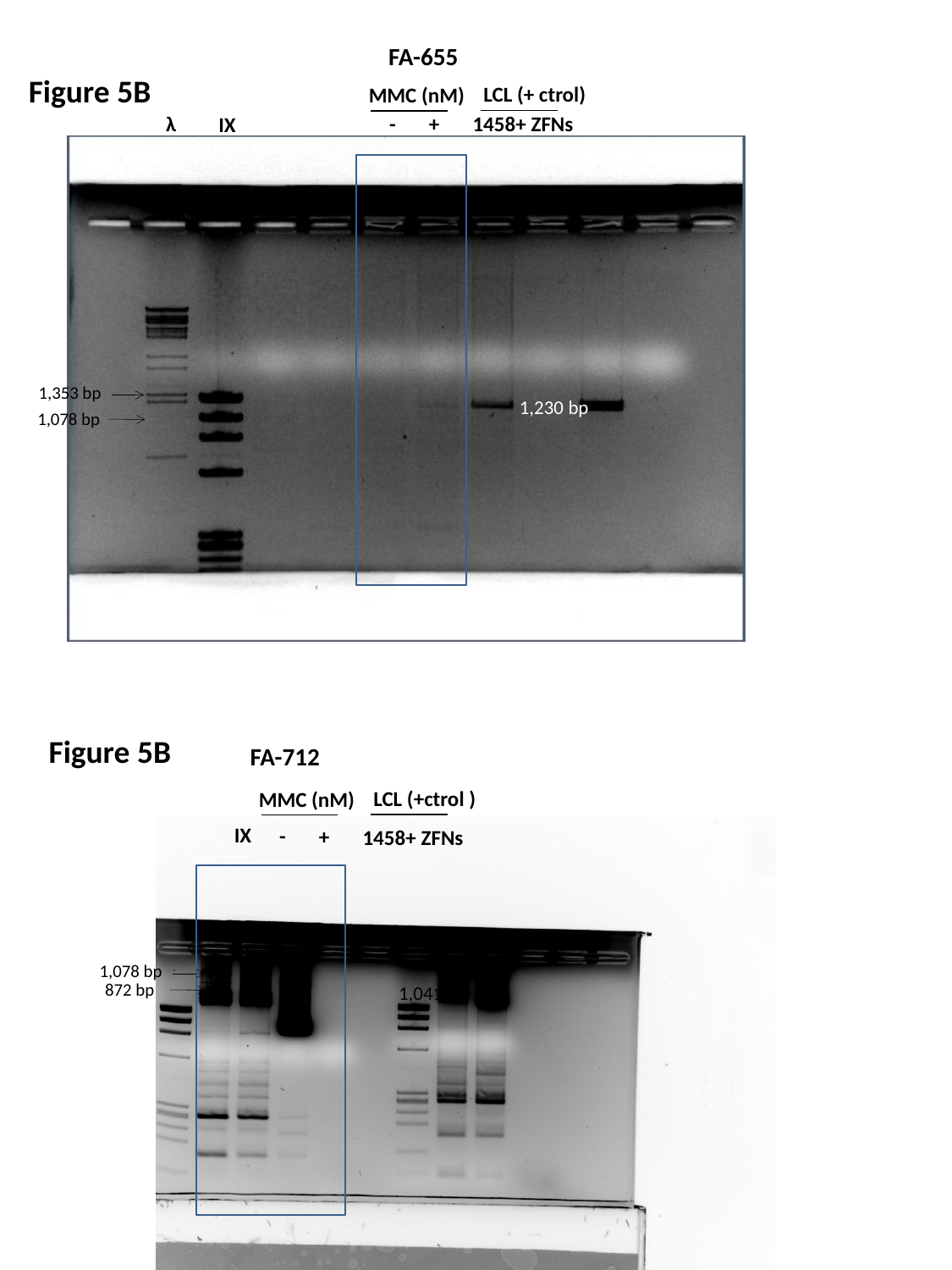

FA-655
Figure 5B
LCL (+ ctrol)
MMC (nM)
λ
-
+
1458+ ZFNs
IX
IX
1,353 bp
1,230 bp
1,078 bp
Figure 5B
FA-712
LCL (+ctrol )
MMC (nM)
IX
-
+
1458+ ZFNs
1,078 bp
872 bp
1,041 bp

## Slide 2
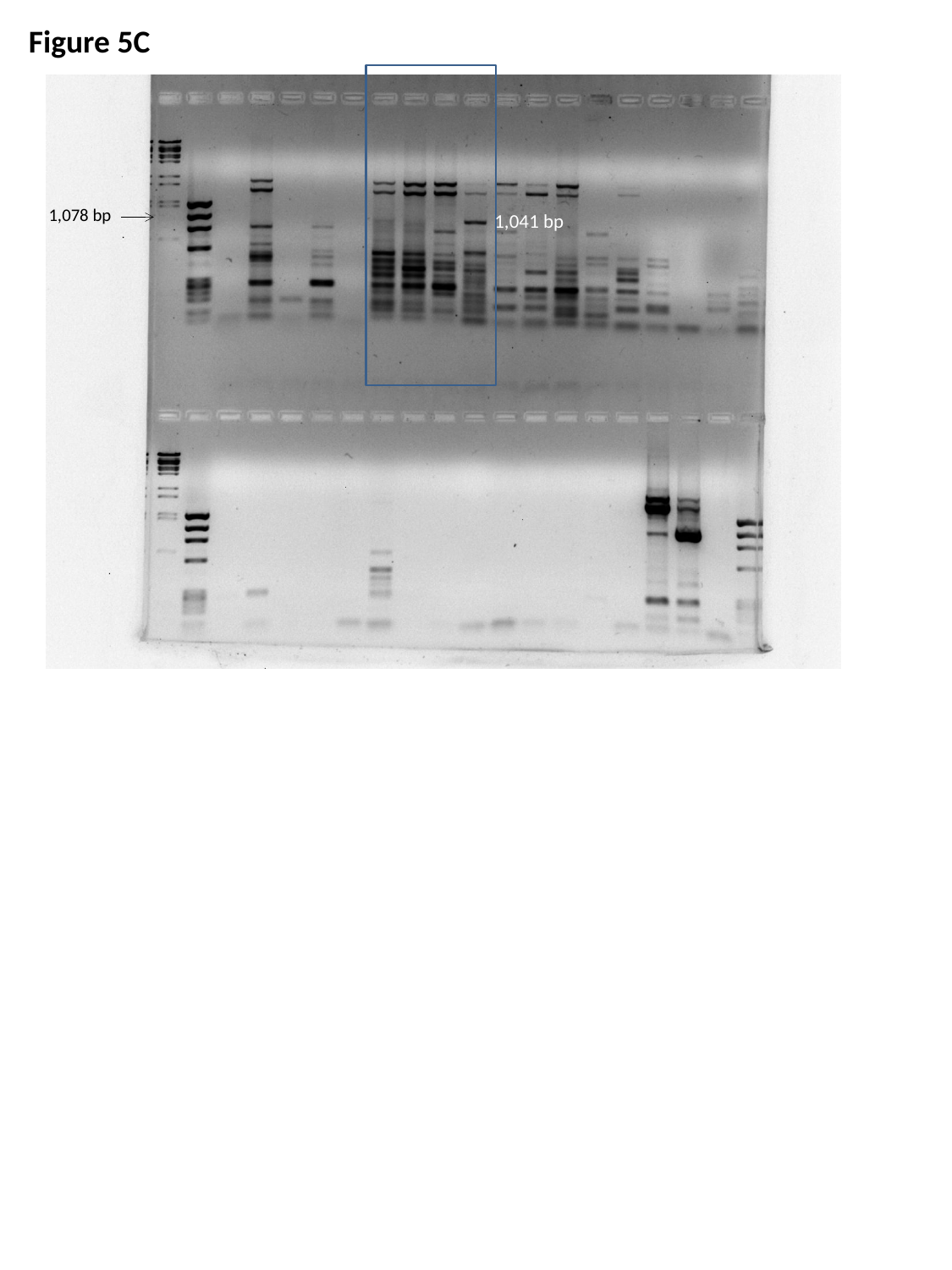

Figure 5C
1,078 bp
1,041 bp
